# Rationally engineered Troponin C modulates *in vivo* cardiac function and performance in health and disease

**DOI:** 10.1038/ncomms10794

**Published:** 2016-02-24

**Authors:** Vikram Shettigar, Bo Zhang, Sean C. Little, Hussam E. Salhi, Brian J. Hansen, Ning Li, Jianchao Zhang, Steve R. Roof, Hsiang-Ting Ho, Lucia Brunello, Jessica K. Lerch, Noah Weisleder, Vadim V. Fedorov, Federica Accornero, Jill A. Rafael-Fortney, Sandor Gyorke, Paul M. L. Janssen, Brandon J. Biesiadecki, Mark T. Ziolo, Jonathan P. Davis

**Affiliations:** 1 Davis Heart and Lung Research Institute and Department of Physiology and Cell Biology, Columbus, Ohio 43210, USA; 2 Bristol-Myers Squibb, Department of Discovery Biology, Wallingford, Connecticut 06492, USA; 3 Q-Test Labs, Columbus, Ohio 43235, USA; 4 Center for Brain and Spinal Cord Repair, Department of Neuroscience, The Ohio State University College of Medicine, Columbus, Ohio 43210, USA

## Abstract

Treatment for heart disease, the leading cause of death in the world, has progressed little for several decades. Here we develop a protein engineering approach to directly tune in vivo cardiac contractility by tailoring the ability of the heart to respond to the Ca^2+^ signal. Promisingly, our smartly formulated Ca^2+^-sensitizing TnC (L48Q) enhances heart function without any adverse effects that are commonly observed with positive inotropes. In a myocardial infarction (MI) model of heart failure, expression of TnC L48Q before the MI preserves cardiac function and performance. Moreover, expression of TnC L48Q after the MI therapeutically enhances cardiac function and performance, without compromising survival. We demonstrate engineering TnC can specifically and precisely modulate cardiac contractility that when combined with gene therapy can be employed as a therapeutic strategy for heart disease.

## Abstract (web summary)

Heart contraction, which is decreased in disease, is determined by Ca^2+^ binding to troponin C. Here, the authors combine a protein engineering approach with gene therapy to modulate heart contractility in mice with the use of rationally designed Troponin C variants, suggesting a new therapy for diseased hearts.

## Graphical abstract

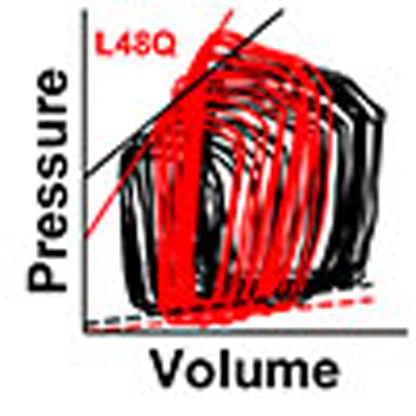

The amount of Ca^2+^ bound to Troponin C (TnC), the Ca^2+^-dependent switch in the heart, is a primary determinant of the strength of cardiac contraction^1^,^2^. Hence, modulating contraction through TnC has been an enticing pharmaceutical goal for many decades. Unfortunately, there are no specific therapeutics that modulate TnC without affecting other systems (for example, phosphodiesterases and hypotension)^3^,^4^. A promising strategy to solely modulate the Ca^2+^ sensitivity of TnC is through rationally customizing Ca^2+^ binding to TnC (refs 5, 6). By applying the principles that govern Ca^2+^ binding to TnC, we have smartly formulated TnC variants with a wide range of Ca^2+^ sensitivities^5^,^6^. These variants effectively modulate Ca^2+^ sensitivity and force development from *in silico* to *in vitro*^5^,^7^,^8^,^9^,^10^,^11^. Whether TnC engineering can translate *in vivo* to modulate cardiac function with molecular precision is currently unknown.

There is a dire need to be able to modulate cardiac contraction to better manage cardiovascular disease. For example, a majority of cardiomyopathies present with impaired contraction^12^. Many strategies to improve cardiac contraction (positive inotropes) do so by increasing the signal for contraction (that is, intracellular Ca^2+^)^13^. Owing to the universal nature of Ca^2+^ signalling, increasing Ca^2+^ not only enhances contraction, but also leads to a variety of negative side effects (arrhythmias and cell death) that ultimately increases mortality^14^. Other inotropic strategies that enhance the sensitivity of the contractile apparatus to Ca^2+^ results in compromised cardiac relaxation and can also raise intracellular Ca^2+^ through off-target interactions^4^. Thus, there is a great need for a positive inotrope that does not have these confounding issues.

We show that our rationally customized TnC can improve function and performance in a common heart pathology–myocardial infarction (MI). We find that our Ca^2+^-sensitizing TnC L48Q expressed before or after an MI, enhances heart function and performance without any adverse effects (that is, slow relaxation, arrhythmias or changing Ca^2+^). Thus, smartly formulating TnC combined with gene therapy is a powerful tool to develop unique and novel therapies with molecular precision to combat heart disease.

## Results

### Engineered TnCs modify cardiac contraction

**Figure 1:**
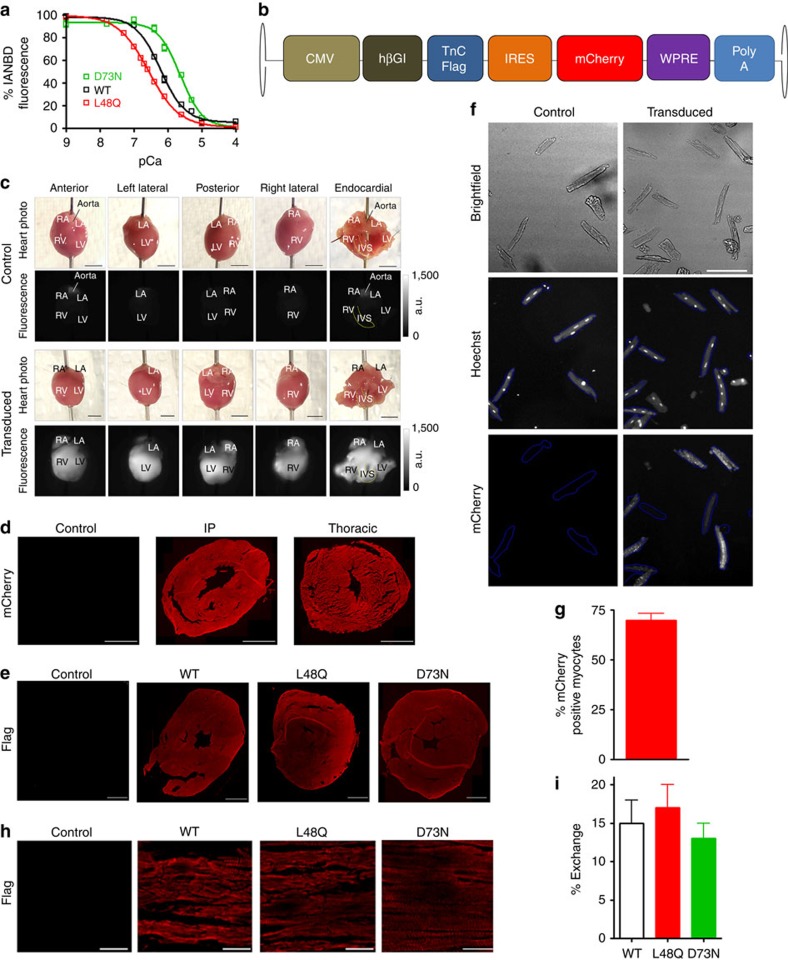
Quantification of rAAV9 transduction efficiency and TnC replacement. (**a**) Summary data of Ca^2+^ sensitivity of rabbit cardiac ventricular myofibrils exchanged with engineered TnC variants. (**b**) Schematic of the gene construct for the expression of the engineered TnC *in vivo* through rAAV9. HβGI: human beta globin intron; IRES: internal ribosome entry site; WPRE: woodchuck hepatitis virus post-transcriptional response element. (**c**) Representative photos (scale bar, 3 mm) and mCherry fluorescence from uninjected (*n*=3) and rAAV9 transduced (*n*=6) hearts. IVS, interventricular septum; LA, left atria; LV, left ventricle; RA, right atria; RV, right ventricle. (**d**) Representative images comparing the rAAV9 transduction of IP versus thoracic cavity injections via histological mCherry fluorescence (scale bar, 1,000 μm). (**e**) Representative images comparing the TnC-FLAG expression via histological anti-FLAG immunofluorescence (scale bar, 1,000 μm). (**f**) Representative images for (**g**) the quantification of the per cent of cardiac myocytes transduced by rAAV9 via high-content microscopy. *n*=2 for control hearts and *n*=3 for rAAV9 transduced hearts (scale bar, 100 μm). (**h**) Representative immunofluorescence images for sarcomeric incorporation of flag-tagged TnC and (**i**) quantification of the per cent TnC exchanged post transduction (samples were pooled and averaged from all the non-diseased and diseased models). Error bars are s.e.m.

We and others have previously shown *in vitro* that the Ca^2+^ sensitivity of force development can be modulated through our rationally engineered TnCs (refs 5, 7, 9, 10, 15). Excitingly Feest *et al*.^7^ recently demonstrated the concept that TnC can enhance contraction in cultured myocytes. To test the efficacy of engineered TnC on *in vivo* cardiac contractility, we delivered either TnC D73N (Ca^2+^ desensitizer) or TnC L48Q (Ca^2+^ sensitizer) into murine myocardium through recombinant adeno-associated virus serotype 9 (rAAV9). TnCs were selected based on their ability to sensitize or desensitize the contractile apparatus to Ca^2+^ in a variety of biochemical and physiological systems (Fig. 1a)^5^,^7^,^9^. The rAAV9 expression cassette contained a cytomegalovirus (CMV) promoter driving the expression of a C-terminally FLAG-tagged TnC variant (Fig. 1b). To track transduction, an internal ribosome entry site-mCherry sequence was added to the vector. We investigated the transduction efficiency of our rAAV9 utilizing several different approaches. At the level of the whole heart, we observed mCherry fluorescence throughout the heart (Fig. 1c and Supplementary Fig. 1) and retrieved 22±8 million vector genomes. Throughout the ventricle, we also observed robust histological mCherry fluorescence in neonatal mice that were injected intraperitoneally (IP) or adult mice injected via the thoracic cavity (to restrict expression to the heart; Fig. 1d and Supplementary Fig. 2). Consistent with these findings, we found expression of Flag-tagged TnC throughout the ventricles (Fig. 1e and Supplementary Fig. 3). Using high-content microscopy to analyse over 3,000 myocytes, we quantified that 70±3 percent of these myocytes were positive for mCherry fluorescence (Fig. 1f,g). This robust transduction of myocytes does not necessarily mean expression or functional incorporation of TnC. Hence, Fig. 1h demonstrates that the transduced TnCs expressed and incorporated into the sarcomere. At this level of myocyte transduction, using quantitative western blot analysis we determined on average 15 percent of the endogenous TnC was replaced by our virally expressed TnCs (Fig. 1i and Supplementary Fig. 4). We also investigated the transduction of other resident cell types in the heart. We observed no transduction of cardiac fibroblasts^16^ and negligible transduction of endothelial and vascular smooth muscle cells (Supplementary Fig. 5a,b). Thus we predict that any functional effects would be due to the transduction of myocytes rather than other cell types.

**Figure 2:**
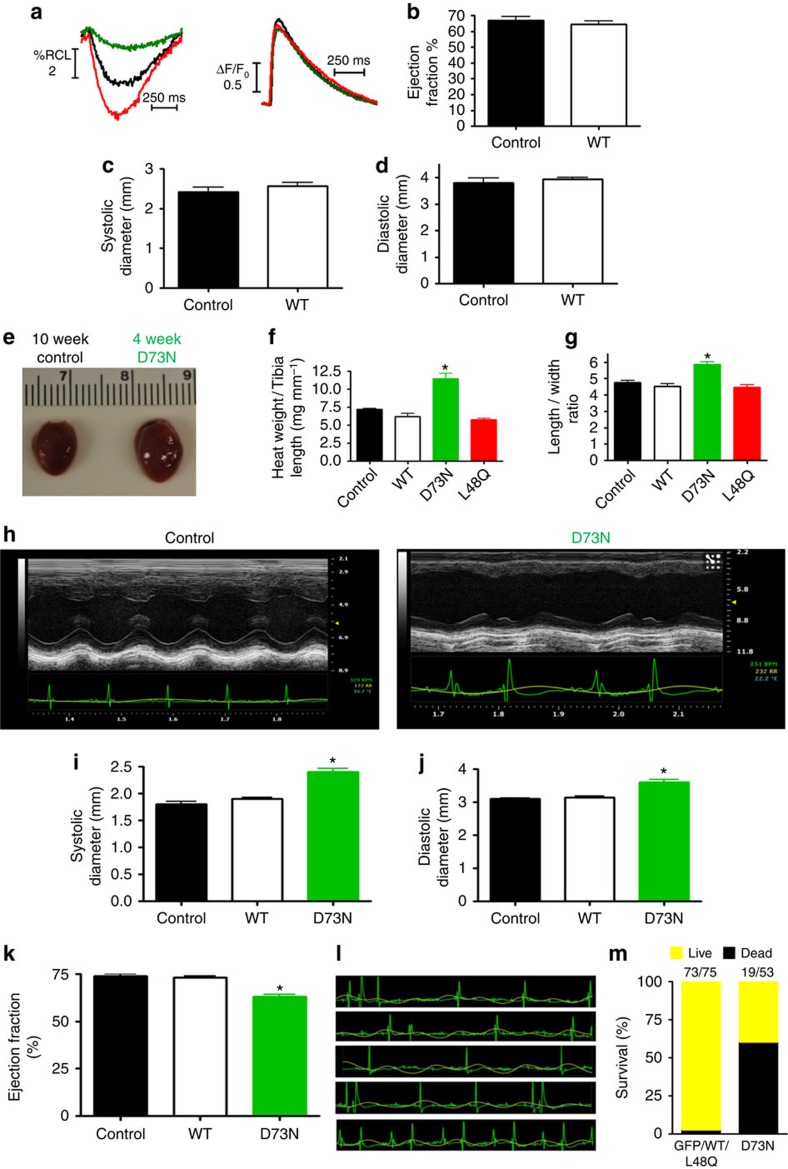
TnC D73N recapitulates a dilated cardiomyopathy. (**a**) Representative traces of isolated cardiomyocyte contraction and Ca^2+^ transients. (**b**) Summary data of TnC WT and control mice EF measured 12 weeks after injection (*n*=4 for control, 5 for TnC WT). Summary data of TnC WT and control mice left ventricular systolic (**c**) and diastolic (**d**) dimensions measured via echocardiography 12 weeks after injection (*n*=4 for control, 5 for TnC WT ). (**e**) Comparison of a 10-week heart from a control mouse and a 4-week heart from a TnC D73N mouse. (**f**) Summary data of heart weight to tibia length (hypertrophy) of rAAV injected mice 4 weeks post injection compared with uninjected (control) mice (*n*⩾ 3 hearts in each group). (**g**) Summary data of isolated cardiomyocyte length–to-width ratio (*n*=25 in each group). (**h**) Representative echocardiography traces from a control and TnC D73N mouse (note the electrical abnormalities in the TnC D73N mouse; lower recordings). (**i**) Summary data of TnC WT, TnC D73N and control mice left ventricular systolic and (**j**) diastolic dimensions measured via echocardiography 4–5 weeks after injection (*n*=5 for control, 4 for TnC WT and 11 for TnC D73N ). (**k**) Summary data of TnC WT, TnC D73N and control mice EF measured 4–5 weeks after injection (*n*=5 for control, 4 for TnC WT and 11 for TnC D73N). (**l**) Representative ECG recordings of arrhythmias observed in a TnC D73N mouse 4 weeks after injection. (**m**) Percentage of surviving mice at 8 weeks post injection. The numbers in the column shows the fraction of total mice alive at 8 weeks after injection. *P<0.05 versus all other groups using analysis of variance and Newman–Keuls pairwise analysis. Error bars are s.e.m.

To determine the cellular influence of modifying TnC Ca^2+^ sensitivity, we isolated ventricular myocytes from the rAAV9 injected mice. Myocytes isolated from the transduced hearts displayed characteristics consistent with altered Ca^2+^ sensitivity. TnC D73N significantly decreased, whereas TnC L48Q increased myocyte shortening compared with TnC wild-type (WT) injected or uninjected (control) myocytes (Fig. 2a, Table 1). The altered shortening was not caused by changes in Ca^2+^ handling (Fig. 2a, Table 1). TnC L48Q had little effect on the rate of myocyte re-lengthening, whereas TnC D73N significantly accelerated re-lengthening (Table 1). There was little effect of D73N or L48Q TnC on contractile kinetics (that is, time to peak shortening; Table 1). Thus, under basal conditions, the engineered TnCs were capable of altering contraction amplitude without affecting the Ca^2+^ signal, similar to the results of Feest *et al*.^7^. Following isoproterenol superfusion, TnC L48Q myocytes demonstrated a normal contractile beta-adrenergic response, while TnC D73N myocytes showed a blunted response (Table 1). Myocytes isolated from mice transduced with WT TnC did not show any difference in myocyte contraction (Table 1) or heart function and structure 12 weeks after injection (Fig. 2b–d).

The depressed myocyte contraction and blunted beta-adrenergic response caused by TnC D73N is indicative of heart disease. In fact, TnC D73N hearts were substantially larger than any of the other injected and uninjected hearts with an abnormal myocyte length-to-width ratio (Fig. 2e–g). Additionally, both the systolic and diastolic dimensions of the left ventricle were enlarged (Fig. 2h–j) and displayed a reduced ejection fraction (EF; Fig. 2k). TnC D73N mice also exhibited abnormal electrical activity (Fig. 2l). Ultimately, after 8 weeks post injection, 65% of the TnC D73N injected animals died (Fig. 2m). Consistent with transgenic modifications that decrease Ca^2+^ sensitivity of cardiac muscle^17^,^18^, virally transduced TnC D73N recapitulates a dilated cardiomyopathy phenotype. In contrast, TnC L48Q, TnC WT, GFP or mCherry injected mice did not show any adverse alteration in morphology, fibrosis, survival (Fig. 2f,g,m and Supplementary Fig. 6) or cardiac function/electrical activities (see below). Consistent with previous studies^18^,^19^,^20^,^21^, functional effects can be accomplished with relatively moderate levels of myofilament protein exchange (Fig. 1i). These findings establish a critical principle that engineering TnC's Ca^2+^ sensitivity can modulate *in vivo* cardiac function.

### TnC L48Q is a promising positive inotrope

**Figure 3:**
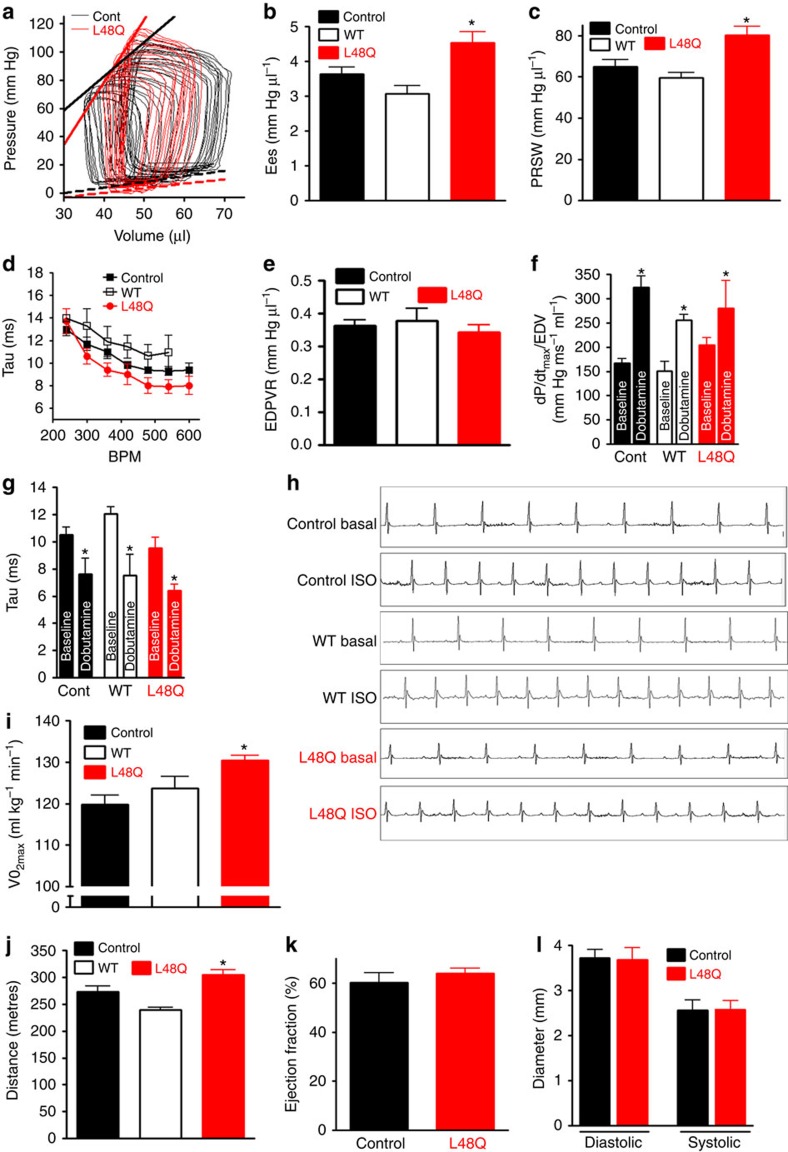
TnC L48Q increases cardiac contractility and performance. (**a**) Representative pressure–volume loops from TnC L48Q and control mice during inferior vena cava occlusion. The thick solid lines are fits for end systolic pressure–volume relationship (ESPVR), whereas the dashed lines are fits for the EDPVR (**b**) Summary data of TnC L48Q, TnC WT and control mice Ees and (**c**) PRSW (*n*=8 for control, 11 for TnC L48Q and 4 for TnC WT). (**d**) Summary data of TnC L48Q, TnC WT and control mice frequency dependent acceleration of relaxation and (**e**) EDPVR (*n*=8 for control, 11 for TnC L48Q and 4 for TnC WT). (**f**) Summary data of TnC L48Q, TnC WT and control mice to beta-adrenergic stimulation (dobutamine, 5 mg kg^−1^) measured as dp/dt_max_/end diastolic volume (EDV) and (**g**) tau. (**h**) Representative ECG traces of anaesthetized mice at baseline and after isoproterenol injection (*n*=6 for TnC L48Q and control, 4 for TnC WT). (**i**) Summary data of VO_2max_ and (**j**) distance run for mice during exercise tolerance testing (*n*=12 for TnC L48Q and control, 6 for TnC WT). Summary data of EF (**k**) and chamber dimensions (**l**) 1 year post injection (*n*=7 for control and TnC L48Q). *P<0.05 versus control (for **f** and **g** *P<0.05 versus corresponding baseline) using two-tailed Student's *t*-test or analysis of variance and Newman–Keuls pairwise analysis as appropriate. Error bars are s.e.m.

In isolated myocytes^7^ (Table 1), TnC L48Q acts as a positive inotrope that does not alter cellular Ca^2+^ or the functional response to beta-adrenergic stimulation, major problems of current inotropes^4^,^14^. To further characterize the potential of TnC L48Q, we performed left ventricular pressure–volume relationship analysis to measure cardiac function *in vivo*^22^. Consistent with the myocyte data, TnC L48Q also significantly enhanced end systolic elastance (Ees) and preload recruitable stroke work (PRSW; Fig. 3a–c), which are load-independent measurements of *in vivo* cardiac contractility^23^. To test if TnC L48Q altered relaxation, as has been observed with various Ca^2+^ sensitizing compounds^4^, we next measured the rate of cardiac relaxation at multiple heart rates along with the end diastolic pressure–volume relationship (EDPVR). The increase in cardiac contractility occurred without delaying the frequency dependent acceleration of relaxation or altering the EDPVR (Fig. 3d,e). Thus, TnC L48Q increases contractility without affecting the diastolic function of the heart.

The major regulator of *in vivo* contractility is sympathetic stimulation^24^. Several of the current positive inotropes increase contraction through the beta-adrenergic pathway^13^. Over stimulating the beta-adrenergic response limits contractile reserve, makes the heart prone to arrhythmias and increases mortality^14^. To test if this pathway is altered in TnC L48Q mice, we investigated the effects of the beta-adrenergic agonist dobutamine. Consistent with our myocyte data, TnC L48Q mice displayed a normal contractile reserve to beta-adrenergic stimulation, again without affecting the ability of the heart to relax (Fig. 3f,g). Another issue with Ca^2+^ sensitization and sympathetic stimulation is the generation of triggered arrhythmias^14^. In conscious, unrestrained mice or anaesthetized mice (±beta-adrenergic agonist isoproterenol), we did not observe electrocardiography (ECG) abnormalities in the TnC L48Q mice that could be associated with atrial or ventricular electrical dysfunction (Fig. 3h, Supplementary Table 1). Furthermore, TnC L48Q did not change cell death or fibrosis (Supplementary Fig. 7). Thus, TnC L48Q increases cardiac function without impairing the beta-adrenergic response, electrical function or morphology.

We next tested whether this increase in cardiac function translated into increased cardiovascular performance. During exercise tolerance testing, a predictor of cardiovascular fitness and health^25^,^26^, the TnC L48Q mice displayed enhanced cardiovascular performance as evidenced by increased VO_2max_ and distance run before exhaustion (Fig. 3i,j). Thus, TnC L48Q has properties of a promising positive inotrope that can increase cardiac function and cardiovascular performance without adversely affecting cardiac morphology, electrical activity, beta-adrenergic response, relaxation, diastolic function, intracellular Ca^2+^ or survival. Unlike other positive inotropes, there appears to be no detrimental impact of long-term (1 year) TnC L48Q transduction on cardiac function, remodelling or survival (Fig. 3k,l). These are the desired characteristics of a positive inotrope that could be used to aid and improve the diseased heart.

### TnC L48Q protects cardiac function and performance after MI

**Figure 4:**
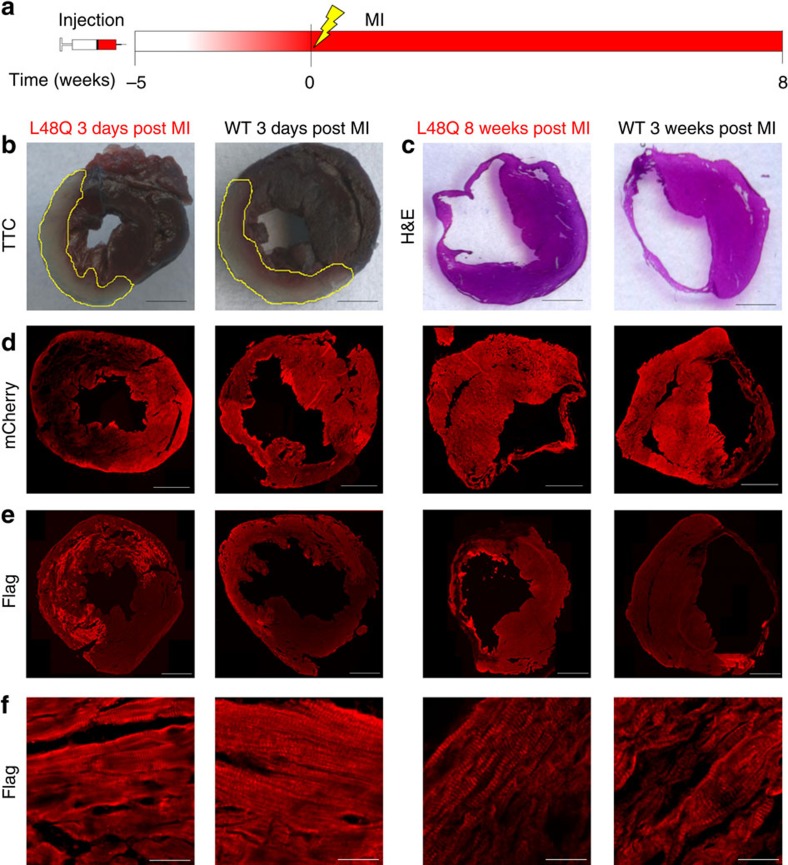
Histological analysis of protective MI hearts. (**a**) Timeline of rAAV9 injection, induction of myocardial infarction and terminal measurements. The gradient of red in the timeline shows the approximate timing of TnC expression. Representative images of infarct size 3 days post-MI (**b**) and 8 weeks post-MI (**c**) for TnC L48Q and TnC WT mice (scale bar, 1,000 μm). Representative images for TnC L48Q and TnC WT-3-days and 8 weeks post MI showing: (**d**) mCherry fluorescence (scale bar, 1,000 μm); (**e**) anti-Flag immunofluorescence (scale bar, 1,000 μm); and (**f**) sarcomeric anti-Flag immunofluorescence (scale bar, 25 μm).

To test if TnC L48Q preserves cardiac function and cardiovascular performance after MI, TnC L48Q and TnC WT expressing mice underwent surgical induction of MI 5 weeks after injection (Fig. 4a). TnC L48Q did not alter infarct size (3 days post MI; Fig. 4b; Supplementary Fig. 8) or reduce scar formation compared with that of TnC WT (8 weeks post MI; Fig. 4c). Even after MI, we observed robust transduction and sarcomeric TnC exchange (Fig. 4d-f).

**Figure 5:**
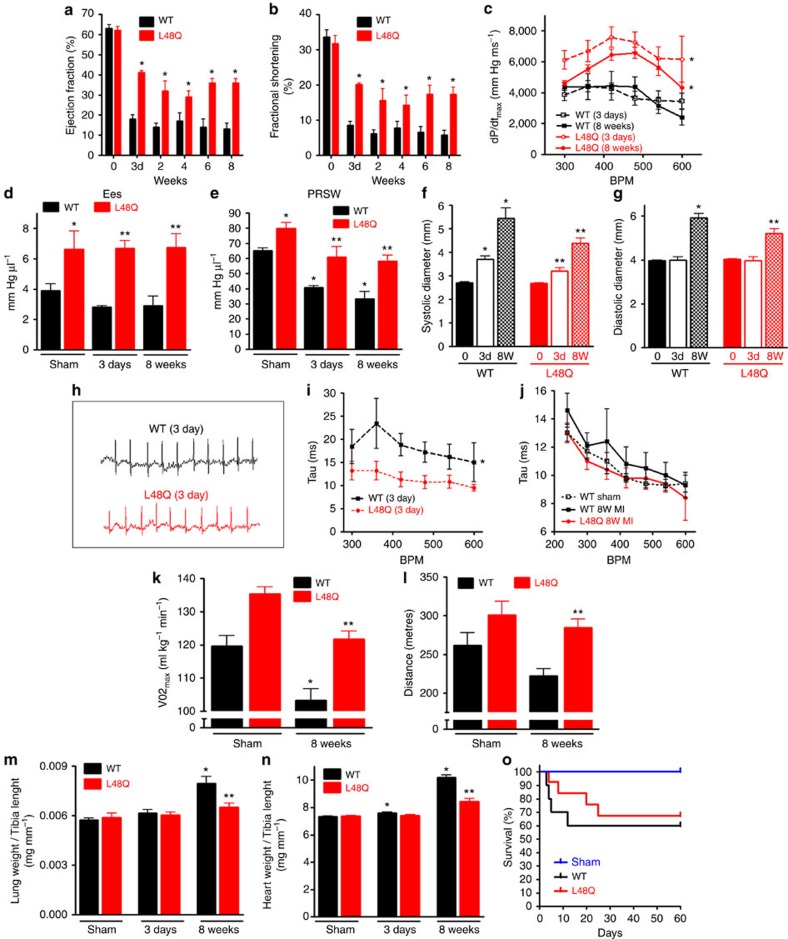
TnC L48Q protects cardiac function and performance after MI. Summary data of sham or before MI (day 0), TnC L48Q and TnC WT mice (**a**) EF and (**b**) fractional shortening before and up to 8 weeks after MI. (**c**) Summary data of dP/dt_max_, (**d**) Ees and (**e**) PRSW 3 days or 8 weeks after MI. (**f**) Summary data of systolic and (**g**) diastolic left ventricular dimensions before, 3 days and 8 weeks post-MI. (**h**) Representative ECG traces in conscious, unrestrained TnC L48Q and TnC WT mice 3 days post-MI. Summary data of frequency dependent acceleration of relaxation (**i**) 3 days and (**j**) 8 weeks post-MI. Summary data of (**k**) VO_2max_ and (**l**) distance run during exercise tolerance testing 8 weeks post-MI. (**m**) Summary data of lung weight to tibia length and (**n**) heart weight to tibia length 3 days and 8 weeks post-MI. (**o**) Kaplan–Meier survival curves (*n*=12 in each group). (For data sets (**a**–**o**) *n*=7 for TnC WT 3 day MI, 9 for TnC L48Q-3 day MI, 6 for TnC WT-8-weeks MI, 8 for TnC L48Q-8 weeks MI,6 for sham TnC WT and 6 for sham TnC L48Q). *P<0.05 versus sham TnC WT, **P<0.05 versus corresponding TnC WT using analysis of variance and Newman–Keuls pairwise analysis. Error bars are S.E.M.

To test the short-term and long-term effects of TnC L48Q, we measured cardiac function beginning at 3 days and up to 8 weeks post MI. Even though >40% of the left ventricle was damaged by MI, TnC L48Q mice were able to resist the MI induced left ventricular dysfunction. At 3 days after MI, TnC L48Q mice presented significantly higher EF and fractional shortening (Fig. 5a,b) and pressure–volume analysis revealed a faster rate of pressure development (dp/dt_max_) across multiple heart rates (Fig. 5c). Moreover, the dp/dt_max_ generated by TnC L48Q myocardium was not significantly different from that of the sham operated mice myocardium (Supplementary Fig. 9). Consistent with increased contractility, Ees and PRSW were also significantly higher in the TnC L48Q mice (Fig. 5d,e). TnC L48Q mice also had lower left ventricular dilation (Fig. 5f,g) and showed no arrhythmia or differences in heart rate variability in conscious and unrestrained TnC L48Q mice (Fig. 5h). Additionally, TnC L48Q mice did not have compromised relaxation (Fig. 5i). Thus, TnC L48Q has positive inotropic properties that provide protective support immediately after MI.

**Figure 6:**
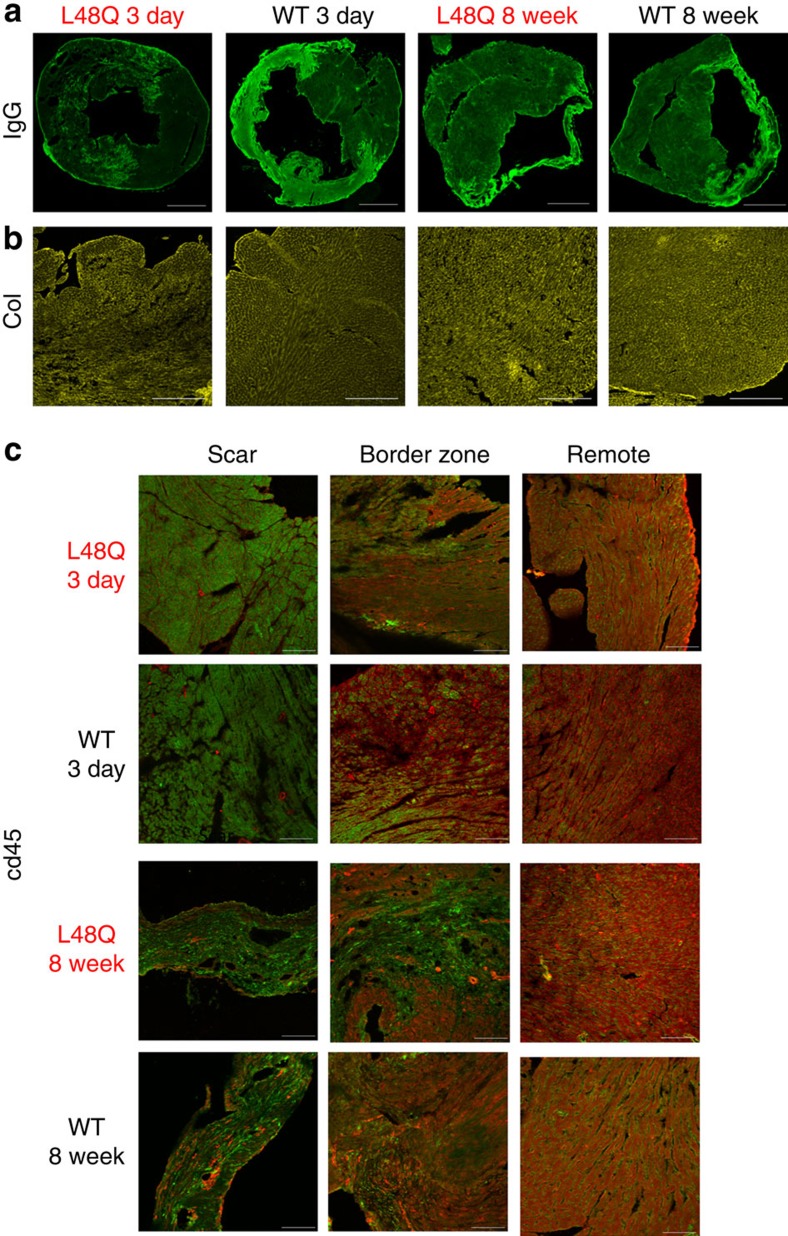
Similar cell death, fibrosis and inflammation after MI in TnC L48Q and TnC WT mice. Representative images of 3 days and 8 weeks post-MI for TnC L48Q and TnC WT mice showing (**a**) IgG immunofluorescence (scale bar, 1,000 μm) and (**b**) fibrosis via collagen immunofluorescence (scale bar, 400 μm). (**c**) Representative images of 3 days and 8 weeks post-MI for TnC L48Q and TnC WT mice showing inflammation of scar, border zone and remote myocardium via CD45 immunofluorescence (green) and mCherry fluorescence (red; scale bar, 100 μm).

Over the 8 week time course, TnC L48Q mice maintained significantly higher EF and fractional shortening (Fig. 5a,b). Pressure–volume analysis demonstrated the significant enhancement of cardiac contractility was also maintained, as evidenced by higher dp/dt_max_, Ees and PRSW (Fig. 5c–e). This increased contractility occurred without impairing the ability of the heart to relax across a wide range of heart rates (Fig. 5j). To test whether this increase in cardiac function after MI translated into increased cardiovascular performance, we subjected the mice to exercise tolerance testing. In line with the maintained cardiac function, TnC L48Q mice displayed higher VO_2max_ and ran a significantly longer distance before reaching exhaustion (Fig. 5k,l). Consistent with protected cardiac function and performance, the lung weight of the TnC L48Q mice did not show signs of congestive heart failure, which was evident in TnC WT mice (Fig. 5m). These findings strongly suggest that TnC L48Q is a promising positive inotrope that can protect cardiac contractility and performance after MI. Furthermore, TnC L48Q long-term expression does not cause any adverse cardiac effects. Specifically, TnC L48Q mice resisted ventricular dilation and hypertrophy (Fig. 5f,g,n). Most importantly, there was no increase in mortality after MI commonly observed by long-term use of other inotropic strategies previously tested^27^,^28^ (Fig. 5o). All these functional changes were observed without any difference in cell death, fibrosis or inflammation (Fig. 6a–c). Thus, TnC L48Q protects cardiac function and performance after an MI. We believe our MI data is consistent with TnC improving the contractility of the surviving myocytes rather than salvaging dying tissue or decreasing inflammation.

### Therapeutic role of TnC L48Q

**Figure 7:**
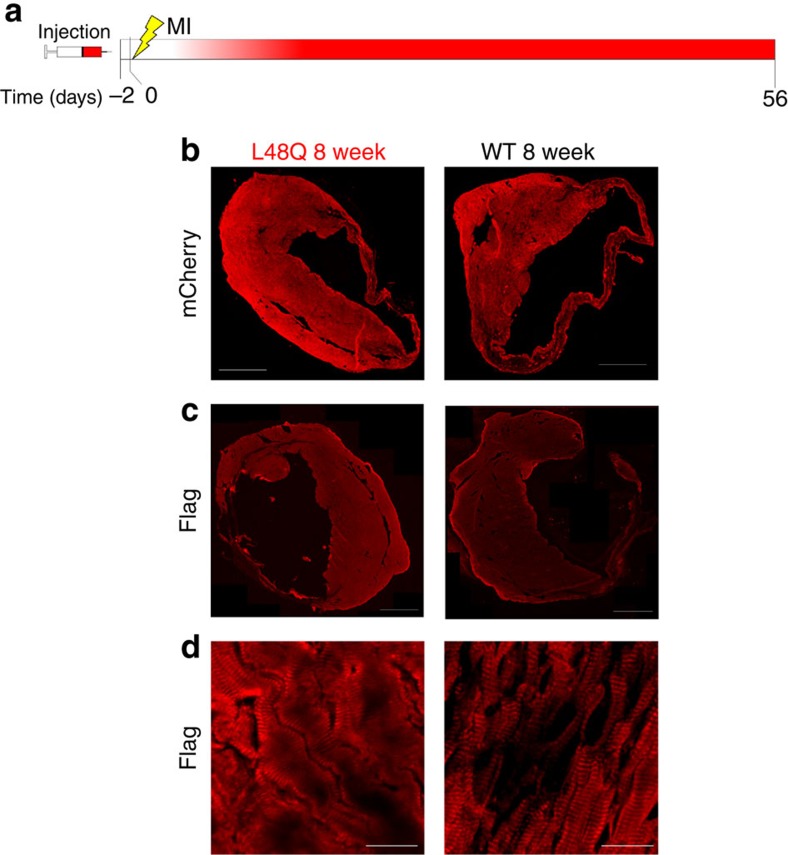
Histological analysis of therapeutic MI hearts. (**a**) Timeline of rAAV9 injection, induction of myocardial infarction and terminal measurements. The gradient of red in the timeline shows the approximate timing of TnC expression. Representative images for TnC L48Q and TnC WT-8-weeks post MI showing: (**b**) mCherry fluorescence (scale bar, 1,000 μm), (**c**) anti-Flag immunofluorescence (scale bar, 1,000 μm); and (**d**) sarcomeric anti-Flag immunofluorescence (scale bar, 25 μm).

**Figure 8:**
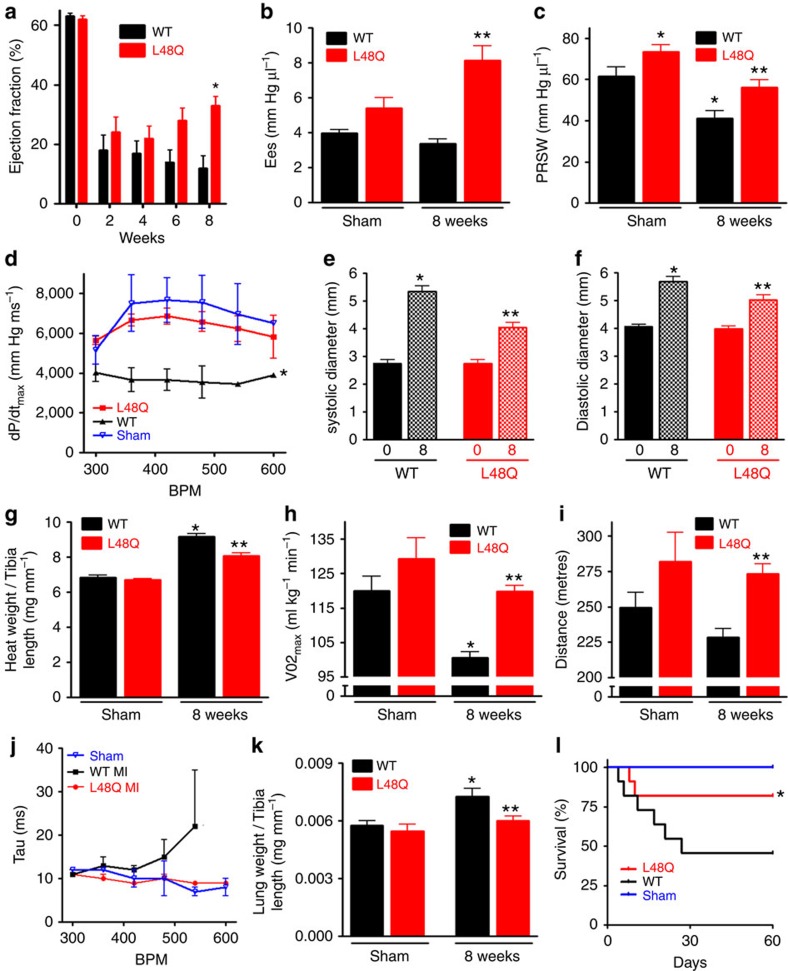
Therapeutic effects of TnC L48Q after an MI. (**a**) Summary data of TnC L48Q and TnC WT mice EF before and up to 8 weeks post-MI (*n*=12 per group). Summary data of (**b**) Ees and (**c**) PRSW, (**d**) dp/dt_max_ 8 weeks post-MI. (**e**) Summary data of systolic and (**f**) diastolic left ventricular dimensions before and 8 weeks post-MI. (**g**) Summary data of heart weight to tibia length 8 weeks post-MI and sham. (**h**) Summary data of VO_2max_ and (**i**) distance run during exercise tolerance testing 8 weeks post-MI and sham. (**j**) Summary data of TnC L48Q and TnC WT mice frequency dependent acceleration of relaxation 8 weeks post-MI and sham. (**k**) Summary data of lung weight to tibia length. (**l**) Kaplan–Meier survival curves. *n*=12 per group. For data sets (**b**-**l**) *n*=5 for TnC WT, 9 for TnC L48Q, 4 for sham TnC WT and 4 for sham TnC L48Q. *P<0.05 versus WT sham, **P<0.05 versus corresponding TnC WT using analysis of variance and Newman–Keuls pairwise analysis. Error bars are s.e.m.

**Figure 9:**
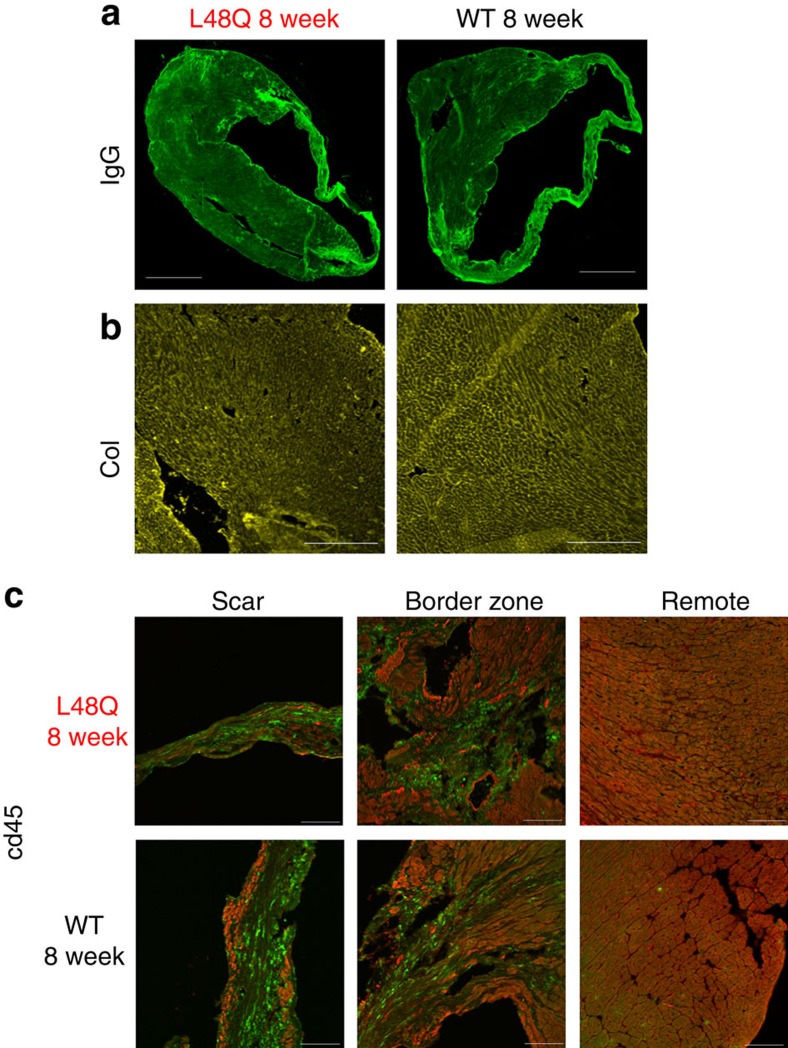
Similar cell death, fibrosis and inflammation after MI in TnC L48Q and TnC WT mice. Representative images for TnC L48Q and TnC WT mice 8 weeks post-MI showing (**a**) IgG immunofluorescence (scale bar, 1,000 μm) and (**b**) fibrosis via collagen immunofluorescence (scale bar, 400 μm). (**c**) Representative images for TnC L48Q and TnC WT mice 8 weeks post-MI showing inflammation of scar, border zone and remote myocardium via CD45 immunofluorescence (green) and mCherry fluorescence (red; scale bar, 100 μm).

To assess the therapeutic potential of TnC L48Q as an inotropic intervention, we modified our experimental strategy so that expression of TnC L48Q would begin ∼1–2 weeks after the infarct (Fig. 7a). Eight weeks after the MI, we observed robust TnC expression and sarcomeric exchange (Fig. 7b–d). Over the 8 weeks (56 days) of observation, TnC L48Q resulted in a steady and significant improvement in EF (Fig. 8a). Pressure–volume analysis at 8 weeks revealed that the TnC L48Q mice had enhanced cardiac contractility (Ees and PRSW; Fig. 8b,c) with dP/dt_max_ comparable to that developed by the sham operated mice (Fig. 8d). The improvement in cardiac contractility resulted in significantly less remodelling (that is, ventricular dilation and hypertrophy, Fig. 8e–g). Consistent with the improved contractility and function, TnC L48Q also increased the exercise tolerance of the mice as demonstrated by significantly enhanced VO_2max_ and distance run before reaching exhaustion (Fig. 8h,i). Furthermore, TnC L48Q mice did not have compromised relaxation and showed no sign of congestive heart failure (Fig. 8j,k). Unlike other positive inotropes, long-term transduction of TnC L48Q lessened mortality (Fig. 8l). In this instance, we observed a larger number of infarcted TnC WT mice die (compared with Fig. 5o). Considering the majority of mice die within 2 weeks after MI, we injected mice with TnC L48Q at this time point and still observed a progressive improvement in EF (Supplementary Fig. 10). All these functional changes were observed without any difference in cell death, fibrosis or inflammation (Fig. 9a–c). These findings demonstrate that TnC L48Q is capable of improving the contractility of the surviving myocytes rather than salvaging dying tissue or decreasing inflammation of an infarcted heart to act as a therapeutic intervention.

## Discussion

Here, we provide the first data that smartly formulating sarcomeric proteins can be used to improve cardiac function and performance *in vivo*. Previous *in vitro* work in simplified physiological systems showed that altering Ca^2+^ binding to TnC has a strong influence on contraction^9^,^15^. However, in these systems, Ca^2+^ levels were typically held at steady-state. With the dynamic changes to Ca^2+^ in the *in vivo* heart, it has been presumed that altered Ca^2+^ binding to TnC would be ineffective (that is, TnC would be subservient to the Ca^2+^ signal). However, we believe that the response of the contractile apparatus is finely tuned to the Ca^2+^ signal^2^,^29^,^30^. That is, these systems work in concert to ultimately dictate the level of contraction. Thus, changing either system (the Ca^2+^ trigger or the response) will influence contractility^2^. Our data strongly suggests that altering the Ca^2+^ sensor (TnC) has a significant impact on *in vivo* heart function and performance. Thus, modulating TnC is a logical approach to regulate cardiac contraction in health and a possible therapeutic approach for disease, without the confounding issues of increasing intracellular Ca^2+^.

While generating new myocytes^31^ and/or limiting cell death^32^ (which we did not observe) are viable treatment options, prevailing thought is that positive inotropes are ‘whipping a sick horse to death'^33^ and only used as a last resort (decompensated heart failure)^34^. We are resurrecting an ‘old idea'^1^ that increasing contractility will aid a diseased heart since our approach does not have detrimental effects. Using a model of impaired heart function (MI), the Ca^2+^ sensitized TnC L48Q was able to aid the diseased heart. In fact, the *in vivo* performance of TnC L48Q suggests that it may be a promising positive inotrope. That is, transducing the myocardium with TnC L48Q increases cardiac function and performance without adversely affecting cardiac morphology, electrical activity, beta-adrenergic response, relaxation, diastolic function, intracellular Ca^2+^ or survival. Furthermore, TnC L48Q was not only protective but also therapeutic and beneficial chronically, unlike other positive inotropes^27^. These positive effects can be achieved with only a modest replacement of the endogenous TnC, increasing the likelihood of gene therapy approaches for sarcomeric proteins. Since we did not observe any changes in cell death, fibrosis or inflammation, we propose a new paradigm that smartly formulating TnC may be a viable therapeutic approach for many cardiomyopathies.

On the other hand, there are several cardiomyopathies that manifest with hypercontractility^35^. Our previous *in vitro* work has designed specific TnC constructs to correct these hypercontractile disorders (that is, TnC D73N), as well as restore the aberrant responses that cause different types of familial and acquired cardiomyopathies^5^. Hence, we are able to customize a variety of TnC sensors to tune the contractile response of various cardiac diseases. Combining gene therapy^36^ with designer proteins opens the door for unprecedented personalized medicines against the plethora of diseases.

## Methods

### Animal care and use

All the experiments were performed using C57Bl/6 J mice (Jackson Laboratories). For the intraperitoneal injection studies, a mixed population of males and females were injected at 2–3 days of age and experimented at 4–8 weeks of age. For the intra-thoracic cavity injection studies, male mice were injected at 12–14 weeks of age, and serially studied as indicated. All the mice were housed in an animal facility with a 12-h light/dark cycle and free access to food and water. All experimental procedures were approved and met the guidelines set by the Institutional Animal Care and Use Committee at The Ohio State University.

### Study groups

Control represents non-injected, normal mice. WT represents mice injected with rAAV9 containing a flag-tagged WT TnC. L48Q represents mice injected with rAAV9 containing a flag-tagged L48Q TnC. D73N represents mice injected with rAAV9 containing a flag-tagged D73N TnC.

### Myofibril Ca^2+^ sensitivity

Ventricular cardiac muscle was obtained from male New Zealand White rabbits (2–3 months old)^37^. For obtaining ventricular myofibrils, ventricular muscle was dissected in a Krebs–Henseleit solution (in mM, 137 NaCl, 5 KCl, 1.2 MgSO_4_, 1.2 NaH_2_PO_4_, 20 NaHCO_3_, 10 glucose, 0.25 CaCl_2_ and 20 2,3-butanedione monoxime). Isolated ventricular tissue was minced with scissors in Krebs–Henseleit solution and homogenized with 10 s bursts of a Polytron homogenizer. The suspension was passed through cheesecloth and then further Dounce-homogenized. The myofibrils were collected by centrifugation and resuspended in buffer A (in mM, 10 MOPS, 150 KCl, 3 MgCl_2_, 1 dithiothreitol and 0.02% Tween 20 (pH 7.0)) containing N-((2-(iodoacetoxy)ethyl)-N-methyl)amino-7-nitrobenz-2-oxa-1,3-diazole (IANBD)-labelled Tn (6 μM stock in buffer A) and stored at 4 °C overnight. After the overnight Tn exchange, the myofibrils were washed three times with buffer A to remove the un-exchanged Tn. Steady-state Ca^2+^ sensitivity was performed using a Perkin Elmer LS55 spectrofluorimeter at 15 °C with excitation of 470 nm for IANBD fluorescence. Microliter amounts of CaCl_2_ were added with the [Ca^2+^]_free_ measured using a computer programme developed by Robertson and Potter^6^.

### Virus production and injection

Adeno-Associated Virus serotype 9 was produced in HEK293 cells and purified using an iodixanol gradient purification^38^. The rAAV9 titre was obtained via TaqMan (LifeTechnologies) based quantification using a CMV sequence specific probe. For neonatal mice, 30–50 μl containing 1 × 10^11^ viral genomes were injected at 2–3 days of age through intraperitoneal injection of both male and female pups. Adult (12–14 weeks old) male mice were injected through the intra-thoracic cavity with 100 μl containing 1 × 10^11^ viral genomes. Mice were lightly anaesthetized (1% isoflurane) and kept in supine position with clear access to the chest area. A 29.5 gauge needle was then inserted at an angle halfway between the ribs and ∼7.5 mm left of sternum. Care was taken not to insert the needle into the lungs or heart. We developed this technique to restrict the viral transduction to the adult heart. We chose this technique over straight intracardiac injection because: (1) it's very simple and straightforward; (2) can be performed extremely quickly; (3) no additional instrumentation or surgery is required; (4) it is highly reproducible; (5) requires only a single injection and (6) most importantly, there is significantly less potential for damaging the rapidly beating heart.

### Whole-heart fluorescence imaging

A central pin was placed from the atria through the apex of the heart to standardize rotational positions. The entire epicardial surface of each heart was imaged by consequent 90° rotation around the longitudinal axis to capture emission light from anterior, left lateral, posterior and right arterial projections in a bath of 4 °C Tyrode solution^39^. Next, a cut was made at the anterior ventricular septum to open the right and left ventricles and expose the endocardial surface. Excitation light generated by four halogen lamps was passed through an excitation filter (525±25 nm, BrightLine). The fluorescent light emitted from the heart was bandpass-filtered (585±20 nm, BrightLine) before reaching the MiCAM Ultima-L CMOS camera (SciMedia) with high spatial (100 × 100 pixels, 120 μm per pixel) resolution. The total fluorescent signal from the heart at each projection was averaged, excluding tissue within 0.5 mm of the tissue edge due to light scattering, using customized software (Matlab)^40^.

### Histology

Hearts were frozen in liquid nitrogen cooled isopentane using Optimum Cutting Temperature Compound (OCT) (Tissue-Tek). 8 μm cryosections were performed for histology. Hematoxylin and eosin and triphenyl tetrazolium chloride staining were performed. In brief, the hearts were excised and cut into 2-mm-thick transverse slices. The transverse slices were incubated with 0.75% triphenyl tetrazolium chloride solution (15 min, 37 °C) and fixed in 4% formalin solution (24 h at room temperature). The infarct area was measured using ImageJ (NIH). mCherry fluorescence was obtained directly from that of the virally transduced protein. Immunoglobulin G (IgG) immunofluorescence was performed as a measure of cell death^41^,^42^,^43^,^44^,^45^,^46^ via an anti-mouse IgG goat antibody (1:200, Jackson Immunoresearch). Flag immunofluorescence was performed using a rabbit anti-flag antibody (1:400, Cell Signaling Technology). Collagen immunofluorescence was performed using a rabbit anti-collagen antibody (1:200, Abcam). Endothelial cells were identified via CD31 immunofluorescence using a rat anti-CD31 antibody (1:150, Abcam)^47^. Inflammation was measured via CD45 immunofluorescence using rat anti-CD45 antibody (1:50, Abcam)^48^.

### Qualitative and quantitative expression of exogenous TnC

Samples were prepared by homogenizing mouse ventricular tissue in urea sample buffer (8 M Urea, 2 M Thiourea, 75 mM dithiothreitol, 50 mM Tris-HCl, pH 6.8)^49^.Qualitative detection of rAAV expressed TnC WT from ventricular homogenates was determined via SDS–polyacrylamide gel electrophoresis silver stain. The quantitative amount of rAAV expressed TnC WT or TnC D73N in the cardiac myocyte was determined by western blot^50^. Homogenates were fractionated by SDS–polyacrylamide gel electrophoresis on a 15% (29:1) Laemmli gel and transferred to 0.45 μm polyvinylidene difluoride (PVDF) membrane. Western blot of the transferred membranes was conducted by incubation with the anti-TnC monoclonal antibody 7B9 (Fitzgerald, 1:1000) followed by a fluorescent labelled secondary antibody (Jackson ImmunoResearch, 1:3000) for detection on a Typhoon imager 9410 (GE). The presence of a C-terminal FLAG in the rAAV expressed TnC slows its migration compared with that of the endogenous TnC resulting in distinct exogenous and endogenous TnC Western bands. The TnC D73N band was confirmed via anti-Flag (Sigma, 1:1000). Fluorescent quantification of the 7B9 bands were conducted by ImageQuant TL (GE) in arbitrary units and the amount of exogenous TnC was reported as the per cent exogenous TnC of the total myofilament TnC (for example, exogenous TnC / (exogenous TnC+endogenous TnC) × 100).

We determined that the 7B9 TnC antibody demonstrates ∼10–20-fold decreased affinity to TnC L48Q compared with the exogenous TnC WT or TnC D73N. This was the case for three different commercially available TnC antibodies tested. The decreased antibody affinity against the TnC L48Q likely results from the L48Q amino acid disruption of the TnC antibody epitope that is located in a different region from D73N. Due to the difference in antibody affinity, we were unable to quantify the rAAV exogenous expression of the TnC L48Q by the above method. Instead, using standard curves, we determined the concentration of both the expressed Flag-tagged TnC L48Q and endogenous TnC. To minimize cross-reactivity, parallel gels were run containing identically loaded ventricular samples. The gels also contained a standard curve for known amounts of recombinant Flag-tagged TnC L48Q and control TnC. Anti-TnC 7B9 (Fitzgerald, 1:1000) was used to probe for the endogenous TnC and anti-Flag (Sigma, 1:1000) was used to probe for Flag-tagged TnC L48Q. Based on the standard curves, each tissue sample's endogenous TnC and flag-tagged TnC L48Q was quantified. The per cent of FLAG-tagged TnC L48Q expressed was determined by dividing the concentration of TnC L48Q by the total TnC (μg of expressed Flag-tagged TnC L48Q/(μg of expressed Flag-tagged TnC L48Q+μg of endogenous TnC) × 100). NOTE: Ca^2+^ binding proteins can migrate as two bands dependent upon free Ca^2+^ in SDS^51^. There is different free Ca^2+^ in the heart samples compared to the purified proteins, causing the high affinity TnC L48Q to be most affected.

### Cardiac myocyte isolation and fibroblast culture

Cardiomyocytes were isolated by Langendorff perfusion^52^. Briefly, the heart was cannulated and suspended from the Langendorff apparatus to be perfused with Ca^2+^-free Tyrode solution (Normal tyrode solution in mM: 140 NaCl, 4 KCl, 1 MgCl_2_, 10 glucose, and 5 HEPES, pH 7.4 adjusted with NaOH or HCl) for 5 min. Subsequently, the heart was perfused with a tyrode solution containing Liberase Blendzyme II (0.077 mg ml^−1^) (Roche Applied Science, Indianapolis, IN). After 4–6 min, the heart was removed from the Langendorff apparatus and the ventricles were minced to dissociate the myocyte via trituration. The myocytes were then filtered and centrifuged before being resuspended in a tyrode solution containing 200 μM Ca^2+^. To obtain fibroblasts^53^, the supernatant from the centrifugation step for myocyte isolation was collected and centrifuged at 400 g for 10 min. The pellet obtained was suspended in DMEM media with 10% foetal calf serum and plated on a 6-well tissue culture plate and cultured for 48 h. Media was replaced after 24 h.

### High-content microscopy

Myocytes were plated on laminin coated tissue culture plates and fixed with 4% paraformaldehyde for 20 min at room temperature and then washed with 1XPBS before scanning. The spot detector algorithm (Cellomics ArrayScanXTI, ThermoFisher) was used to identify rod shaped myocytes in the Hoechst channel by adjusting the ObjectShapeLWR parameter. Over 3000 rod shaped myocytes were analysed for quantification of mCherry fluorescence.

### Myocyte contraction/relaxation measurements

Myocytes were loaded with Flou-4 Am (10 μM, Molecular Probes, Eugene, OR) and incubated for 30 min. Myocytes were then washed and allotted an additional 30 min for de-esterification^52^. A Cairn Research Limited (Faversham, UK) epifluorescence system was used for isolated myocyte shortening measurements. Intracellular Ca^2+^ was measured via Fluo-4 epifluorescence with excitation: 480±20 nm and emission: 535±25 nm. The change in fluorescent intensity is expressed as Δ*F*/*F*_0_, where *F* is the fluorescence intensity and *F*_0_ is the fluorescence intensity at rest. Shortening data was collected by video edge detection (Crescent Electronics). Myocytes were field stimulated at 1 Hz via platinum electrodes connected to a Grass Telefactor S48 stimulator (West Warwick, RI).

### Myocardial infarction and Echocardiography

Permanent MI surgery was performed by anaesthetizing mice with isoflurane (2%) and mechanical ventilation. After a left thoracotomy, the fourth intercostal space and the lungs were retracted. The left anterior descending coronary artery was identified under a microscope and permanently ligated with a 8-0 silk suture near its origin. Ligation of the artery was confirmed by distal palor of the left ventricular anterior wall. The sham group had similar surgical procedure without tightening of the suture around the artery. The mice were kept on heat pad until recovery of consciousness. All mice were observed for the next 72 h after surgery. Echocardiography was performed using a VEVO 2100 Visual Sonics (Visual Sonics, Toronto) system^54^. The mice were lightly anaesthetized (1.5% isoflurane) and the EF, fractional shortening and ventricular chamber dimensions were determined through M mode using the parasternal short axis view.

### Pressure–volume loop

Cardiac haemodynamic measurements were assessed via a closed chest approach using a 1.4 French Millar Pressure catheter (AD Instruments) advanced into the left ventricle through the right carotid artery^22^. In brief, mice were anaesthetized by ketamine (55 mg kg^−1^) plus xylazine (15 mg kg^−1^) in saline solution and placed in supine position on a heat pad. Following a midline neck incision, the underlying muscles were pulled to expose the carotid artery. Using a 4-0 suture the artery was tied and the pressure–volume catheter was advanced through the artery into the left ventricle of the heart. After 5–10 min of stabilization, values at baseline and stimulation at varying frequencies (4–10 Hz) were recorded. Pressure–volume loops were also obtained at varying preloads via inferior vena cava occlusions to get Ees, PRSW and EDPVR. To measure the beta-adrenergic response, 5 mg kg^−1^ dobutamine was injected intraperitoneal. All the measurement and analysis were performed on LabChart7 (AD Instruments).

### ECG

In unanaesthetized, unrestrained mice, the electrocardiogram was measured using an ECGenie system (Mouse Specifics, Inc.)^43^. Mice were placed on a footplate electrode for 30 min and ECG was recorded. For anaesthetized ECG recording^55^, mice were anaesthetized using 2% isoflurane and placed in prone position on a heating pad to maintain body temperature. Subcutaneous electrodes were placed in the Lead II position and baseline ECG was measured for 5 min on Powerlab 4/30 (AD Instruments). After 5 min, isoproterenol was infused intra-peritoneally to measure ECG under beta-adrenergic stimulation for 15 min.

### Exercise tolerance testing

A metabolic chamber with treadmill and Oxymax analyzer (Columbus Instruments) were used to measure VO_2max_ and distance run^54^. The mice were acclimatized at a lower speed (6 m min^−1^ at 0^o^ incline) for 10 min a day before the actual measurement. After a 5-min initiation period, the treadmill was adjusted to a 6-m min^−1^ walk for 5 min. The treadmill was then placed at a 20^o^ incline and the speed increased 1 m min^−1^ every minute until the mice reached exhaustion (mice unwilling to run and neglecting an electrical shock). VO_2max_ was defined as the absolute maximal value obtained during the procedure.

### Statistical and data analysis

Sample size was calculated by power analysis using Minitab 5.1. Data exclusion was determined via the Grubbs' test. Investigators collecting and/or analysing the data were blinded to which study group the mouse/tissue belonged. The survival analysis was performed by log-rank test. All other statistical significance was determined by Student's *t*-test or analysis of variance followed by a Newman–Keuls *post hoc* test. All values are expressed as a mean±s.e.m. For all statistical analysis the level of significance was set at *P*<0.05.

## Additional information

**How to cite this article:** Shettigar, V. *et al*. Rationally engineered Troponin C modulates *in vivo* cardiac function and performance in health and disease. *Nat. Commun.* 7:10794 doi: 10.1038/ncomms10794 (2016).

## Supplementary Material

Supplementary Information Supplementary Figures 1-10 and Supplementary Table 1

## Figures and Tables

**Table 1:** Summary data of isolated cardiomyocyte contraction and relaxation under basal conditions and beta-adrenergic stimulation (isoproterenol, 10^−6^ M).

	**Contraction**				**Relaxation**	
	**%RCL**	**TTPs**	**ΔF/F_0_**	**TTPc**	**Shortening RT50**	**Ca Transient RT50**
*Basal*						
Control	3.5±0.4	344±7	1.4±0.1	107±2	336±11	240±7
WT	3.5±0.4	355±7	1.6±0.1	108±2	340±8	241±5
D73N	2.4±0.2*	336±9	1.6±0.1	99±2	302±11*	225±7
L48Q	4.7±0.4*	332±7	1.5±0.1	112±2	326±11	237±9

*ISO*						
Control	7.4±0.9	212±7	4.8±0.3	93±2	192±10	106±4
WT	8.3±1.4	228±7	4.7±0.2	99±2	218±18	119±4
D73N	4.9±0.7*	219±9	3.3±0.3*	91±2	184±12	107±4
L48Q	9.6±1.2	221±5	4.3±0.2	104±2	197±15	123±4

ANOVA, analysis of variance; F/F_0_, Ca^2+^ transient amplitude; %RCL, per cent change in resting cell length; RT50, relaxation time to 50%; TTPc, time to peak Ca^2+^ transient; TTPs, time to peak shortening.

*n*⩾19 myocytes/group.

^*^P<0.05 versus other groups using ANOVA and Newman–Keuls pairwise analysis.
