# Supplementary material for: Rationally engineered Troponin C modulates in vivo cardiac function and performance in health and disease
Source: Nat Commun. 2016 Feb 24;7:10794. doi: 10.1038/ncomms10794 (PMC4770086; doi:10.1038/ncomms10794)
Supplement: Supplementary Information — Supplementary Figures 1-10 and Supplementary Table 1 [file ncomms10794-s1.pdf]

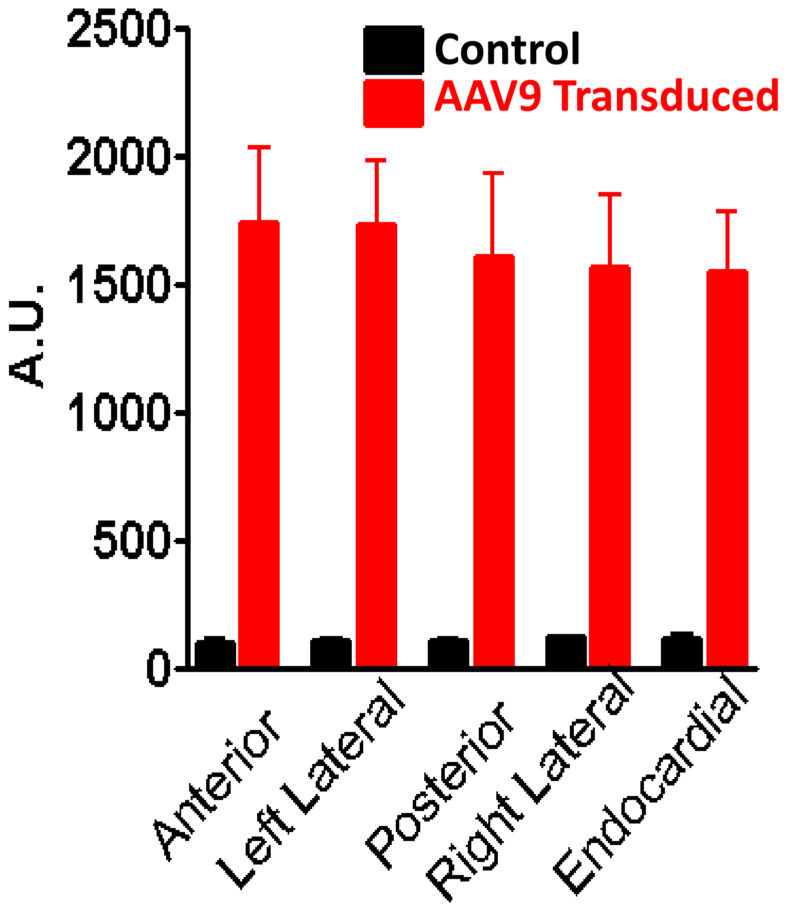

Supplementary Fig. 1. Quantification of mCherry fluorescence intensity across different regions of the whole heart.

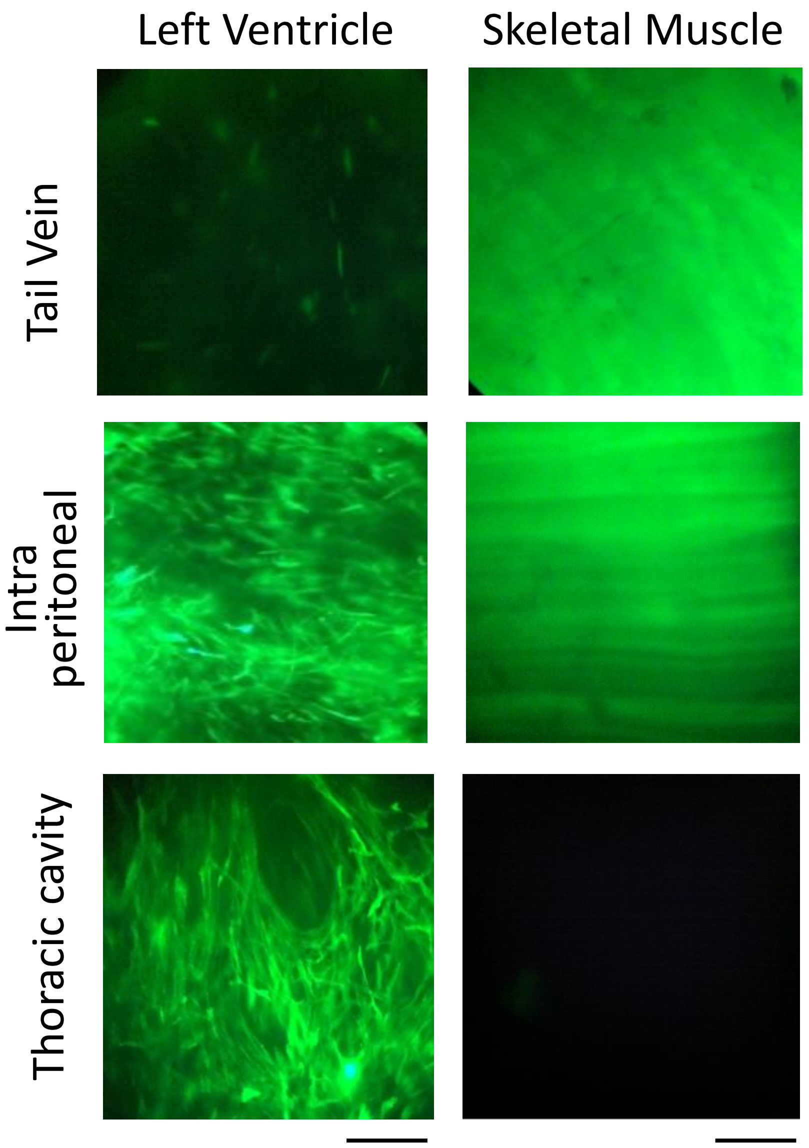

Supplementary Fig. 2. Representative images of AAV9 GFP transduced cardiac muscle (left ventricle) and skeletal muscle (gastrocnemius) using tail vein (top), intraperitoneal (middle) and thoracic cavity injections. The scale bars represent 100µm.

Supplementary Fig. 3. Silver Stained SDS-PAGE of AAV9 TnC WT injected and non-injected ventricular homogenates. Arrows denote the presence of an additional protein band in TnC WT injected ventricle that is not present in non-injected ventricle. The MW of this additional band is consistent to that of FLAG-TnC detected by Western blot (see Figure S4) indicating the expression of FLAG-TnC in the injected ventricle.

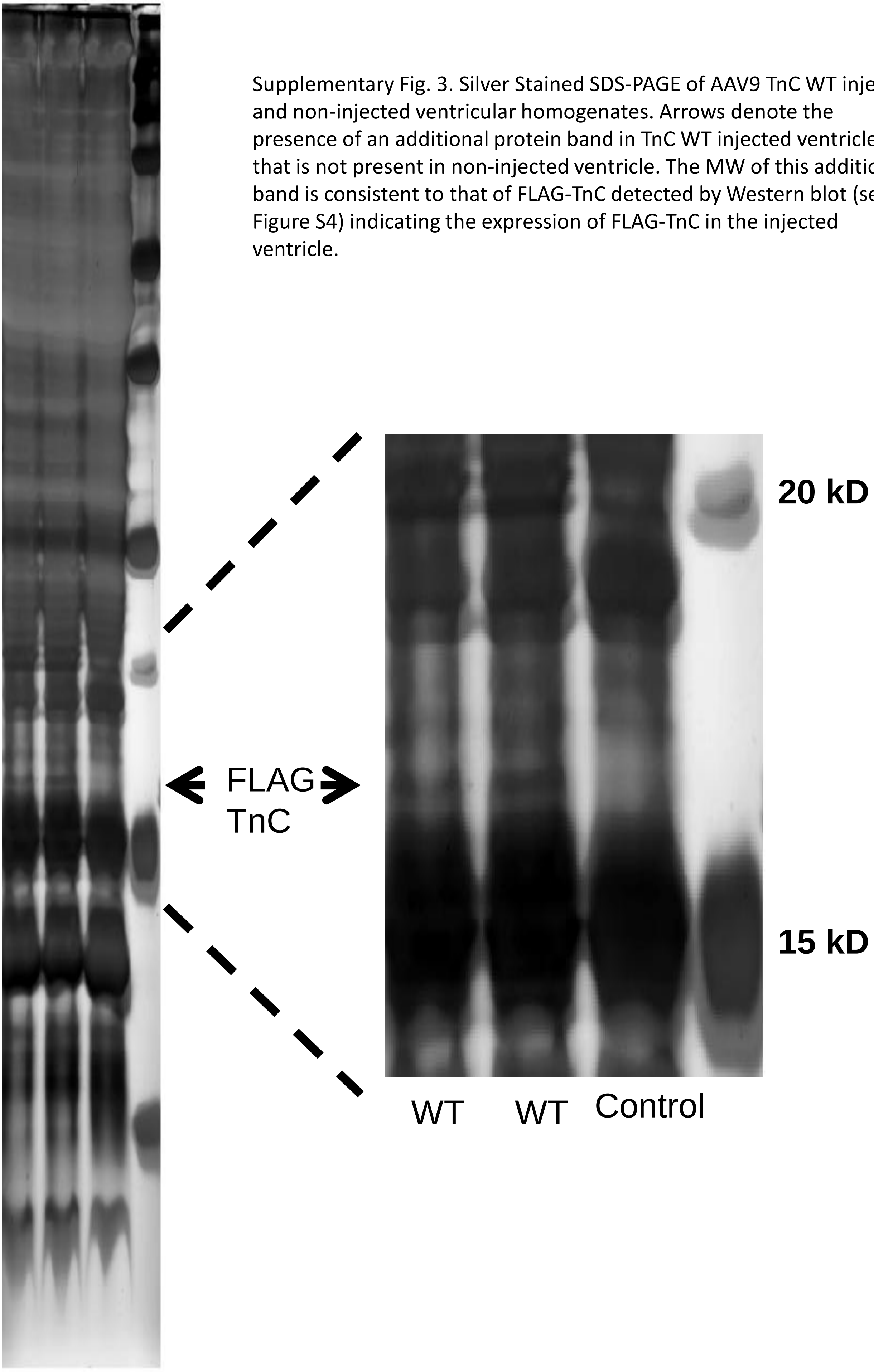

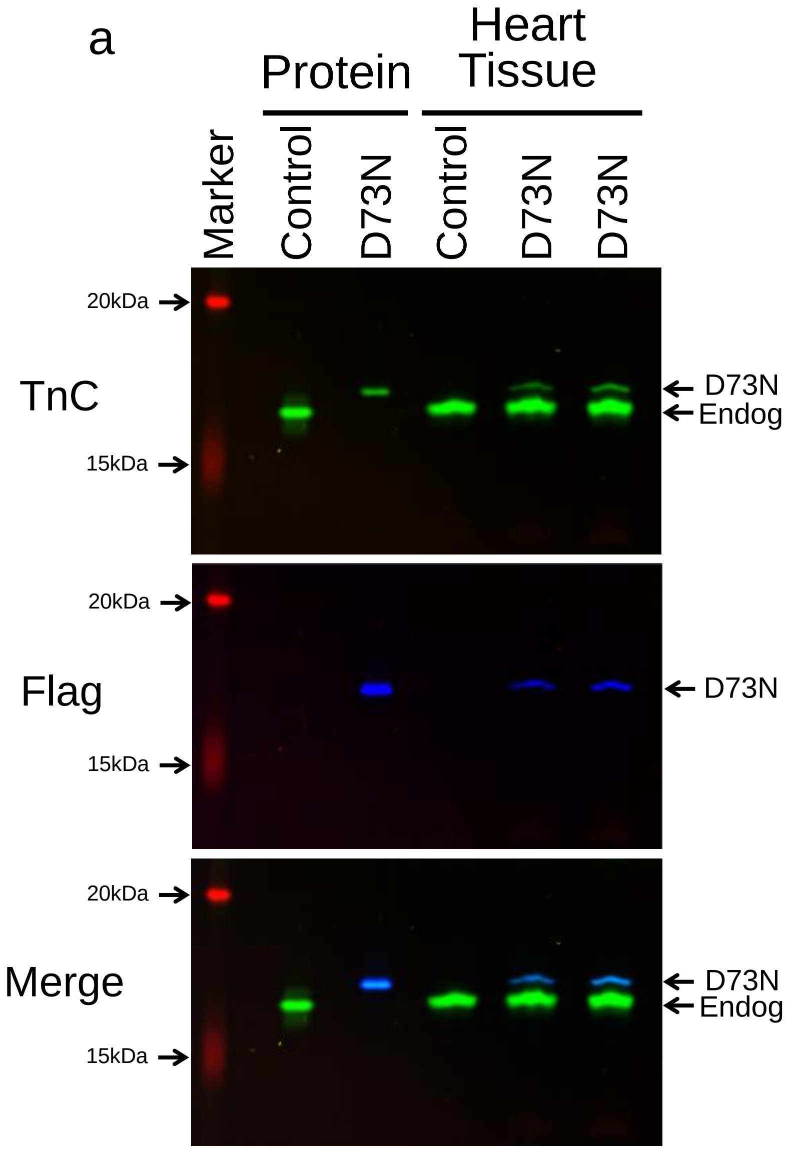

Supplementary Fig. 4. Quantification of Exogneous TnC Expression. (A) Representative western blot for TnC D73N and endogenous (Endog) was probed with the TnC antibody (Top) to calculate the percent expression. To determine which band was TnC D73N the same membrane was probed with the Flag antibody (Middle). Merging the two images demonstrated that while the TnC antibody (Green) recognizes both bands, only the top, slower migrating band was recognized by the Flag antibody (Blue) indicating the top band is TnC D73N and bottom band is Endog TnC. (B) Representative Western blot for Endog TnC with the TnC antibody (Left) and FLAG-tagged TnC L48Q by Flag antibody (Right) was carried out on parallel gels containing samples from two injected mice (heart 1 and heart 2). Each gel also contained increasing known amounts of a TnC standard. NOTE: Ca^{2+} binding proteins can migrate as two bands dependent upon free Ca^{2+} in SDS. There is different Ca^{2+} in the heart samples compared to the purified proteins, causing the higher affinity TnC L48Q to be most affected.

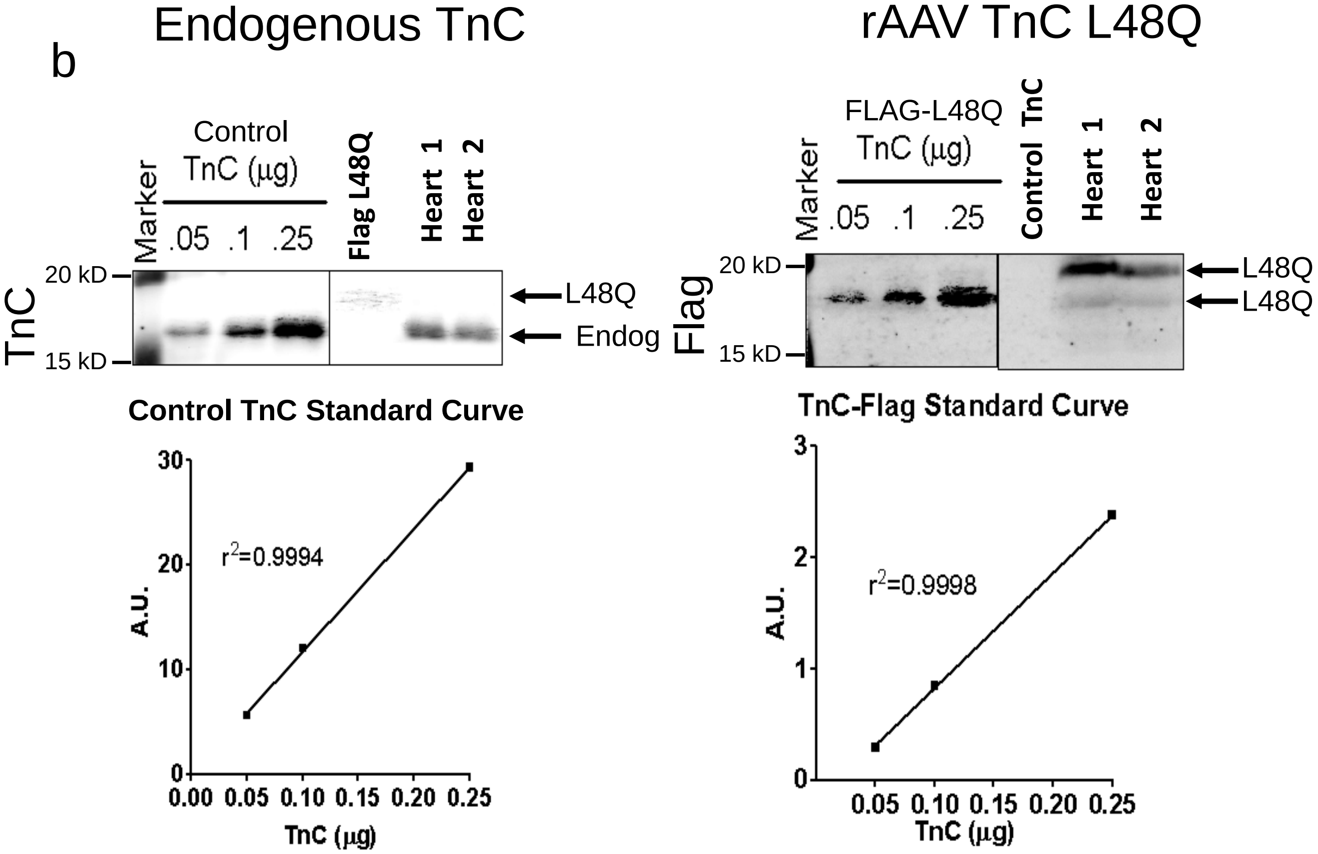

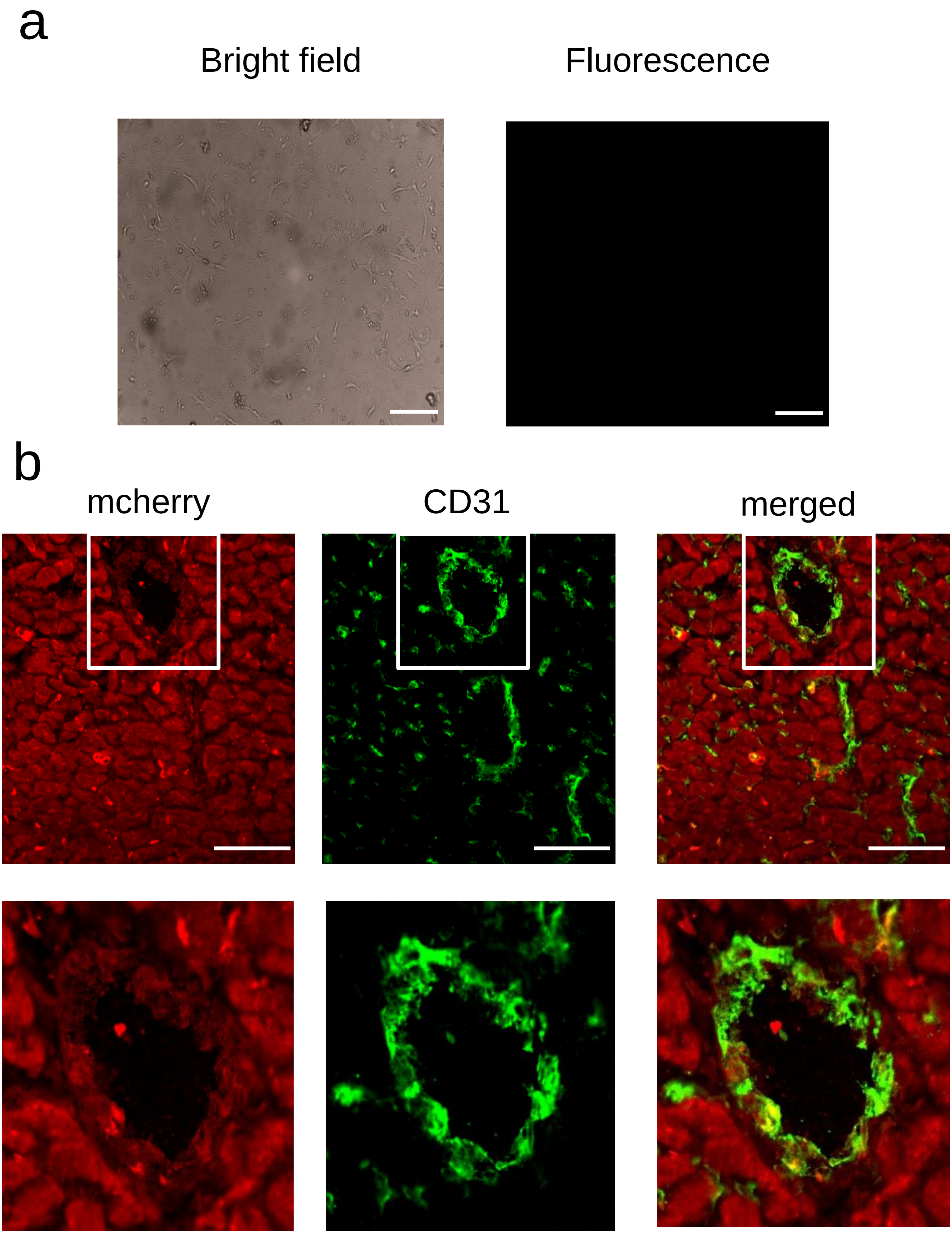

Supplementary Fig. 5. (a) Primary fibroblast culture from a AAV9 transduced heart showing brightfield and mCherry fluorescence. The scale bars represent 100µm. (b) Representative images of AAV9 transduced ventricular histological sections showing (left) mCherry fluorescence, (middle) CD31 positive endothelial cells and (right) merged images. The white box in the top panels are enlarged below. Vascular smooth muscle cells are directly adjacent to CD31 positive cells. The scale bars represent 50µm.

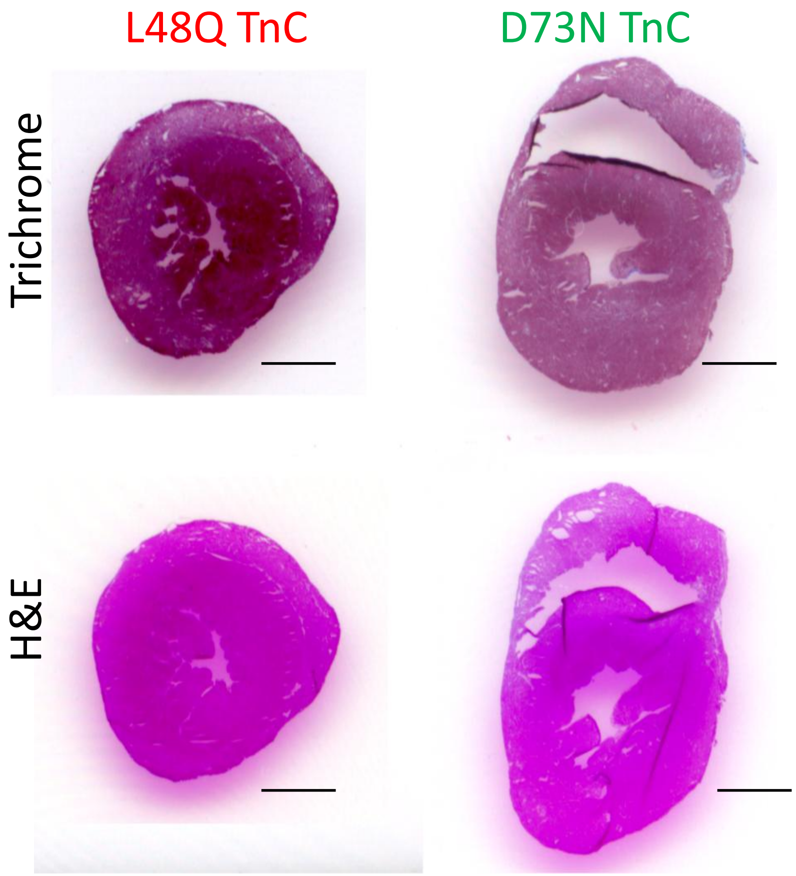

Supplementary Fig. 6. Comparison of H&E and Masson's Trichrome staining between L48Q TnC and D73N TnC expressing mice 4 weeks after AAV9 injection. The scale bar is 1mm.

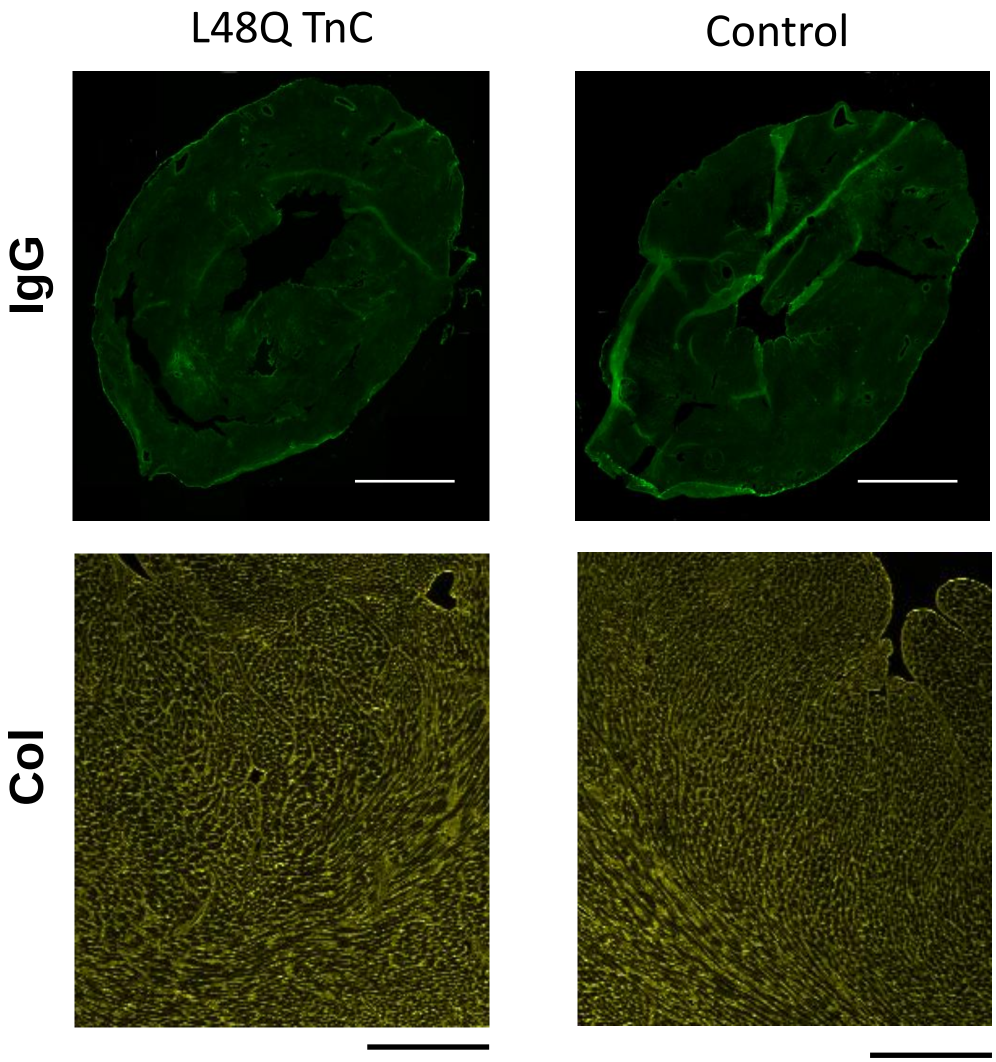

Supplementary Fig. 7. Representative histological sections for IgG and collagen immunohistochemistry from control and TnC L48Q injected hearts. The scale bars represent 1000µm for IgG and 400µm for collagen (Col) images.

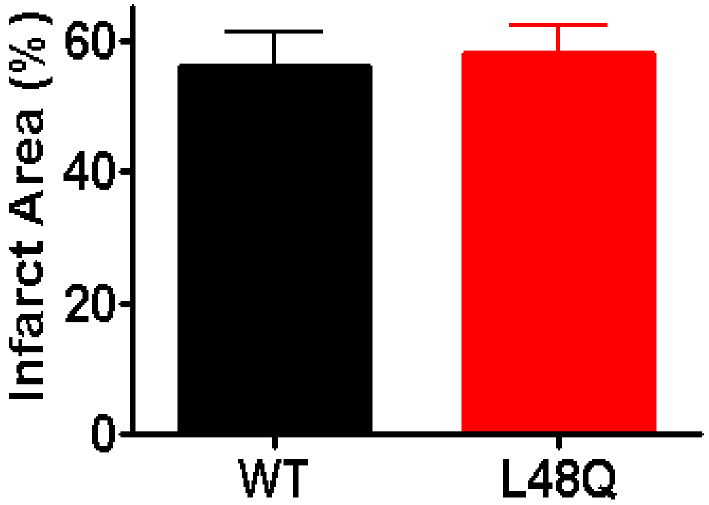

Supplementary Fig. 8. Summary data of left ventricular infarct area in WT TnC and L48Q TnC mice 3 days post-MI.

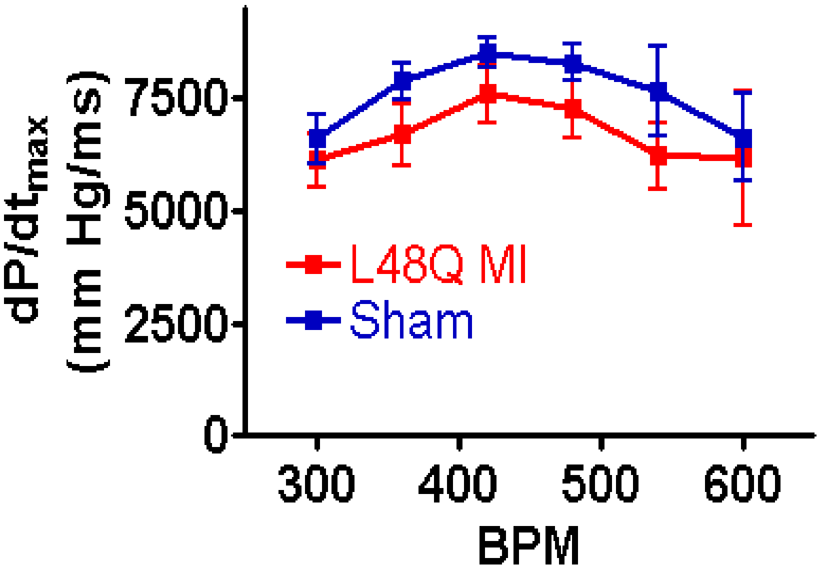

Supplementary Fig. 9. Comparison of dP/dT$_{max}$ between sham operated and L48Q TnC expressing mice 3 days after MI.

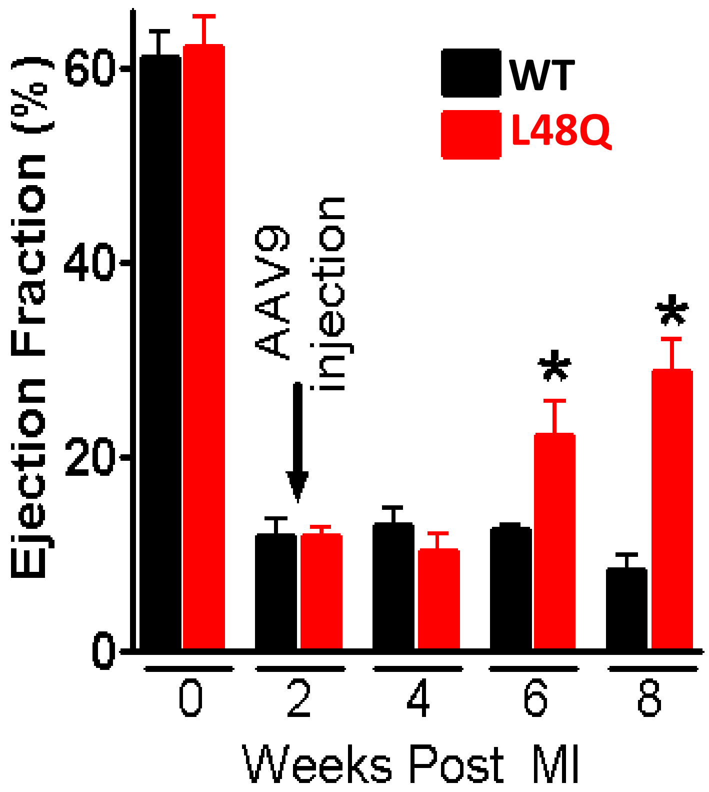

Supplementary Fig. 10. Comparison of ejection fraction for therapeutic MI when the mice were injected 2 weeks post-MI with either TnC l48Q or TnC WT.

	**Heart rate (BPM)**	**Heart rate variability (BPM)**	**QTc (ms)**
control	629±26	74±23	47±2
WT	615±15	58±9	44±2
L48Q	626±30	70±8	48±1

Supplementary Table 1. Summary data of heart rate variability and QT_c in conscious, unrestrained TnC L48Q, TnC WT and control mice (n= 8 for TnC L48Q and control, 4 for TnC WT).
